# Gut microbiome associated with chemotherapy-induced diarrhea from the CapeOX regimen as adjuvant chemotherapy in resected stage III colorectal cancer

**DOI:** 10.1186/s13099-019-0299-4

**Published:** 2019-04-30

**Authors:** Zuo Fei, Yin Lijuan, Yang Xi, Wu Wei, Zhong Jing, Da Miao, Han Shuwen

**Affiliations:** 1 Department of Gastroenterology, Huzhou Central Hospital, No. 198 Hongqi Road, Huzhou, 313000 Zhejiang China; 2 Department of Rheumatology & Immunology, Huzhou Central Hospital, No. 198 Hongqi Road, Huzhou, 313000 Zhejiang China; 3 Department of Intervention and Radiotherapy, Huzhou Central Hospital, No. 198 Hongqi Road, Huzhou, 313000 Zhejiang China; 4 Department of Central Laboratory, Huzhou Central Hospital, No. 198 Hongqi Road, Huzhou, 313000 Zhejiang China; 5 Medical College of Nursing, Huzhou University, No. 759 Erhuan East Road, Huzhou, 313000 Zhejiang China; 6 Department of Medical Oncology, Huzhou Central Hospital, No. 198 Hongqi Road, Huzhou, 313000 Zhejiang China

**Keywords:** Microbiome, Diarrhea, Chemotherapy, Colorectal cancer, CapeOX

## Abstract

**Background:**

Chemotherapy induced diarrhea (CID) is a common side effect in patients receiving chemotherapy for cancer. The aim of our study was to explore the association between gut microorganisms and CID from the CapeOX regimen in resected stage III colorectal cancer (CRC) patients.

**Results:**

After screening and identification, 17 stool samples were collected from resected stage III CRC patients undergoing the CapeOX regimen. Bacterial 16S ribosomal RNA genes was sequenced, and a bioinformatics analysis was executed to screen for the distinctive gut microbiome and the functional metabolism associated with CID due to the CapeOX regimen. The gut microbial community richness and community diversity were lower in CID (p < 0.05 vs control group). *Klebsiella pneumoniae* was the most predominant species (31.22%) among the gut microbiome in CRC patients with CID. There were 75 microorganisms with statistically significant differences at the species level between the CRC patients with and without CID (LDA, linear discriminant analysis score > 2), and there were 23 pathways that the differential microorganisms might be involved in.

**Conclusions:**

The gut microbial community structure and diversity have changed in CRC patients with CID. It may provide novel insights into the prevention and treatment of CID.

**Electronic supplementary material:**

The online version of this article (10.1186/s13099-019-0299-4) contains supplementary material, which is available to authorized users.

## Introduction

Colorectal cancer (CRC) is the third most common malignant tumor worldwide [1]. With the development of early diagnosis methods and molecular targeted therapy, the progression free survival (PFS) and the overall survival (OS) of patients with CRC have increased [2]. However, metastatic colorectal cancer (mCRC) is associated with a poor prognosis [3, 4]. Chemotherapy is a strongly recommended therapeutic regimen to prevent and control mCRC, and it has been associated with a prolonged life [5, 6]. Adjuvant chemotherapy may confer a survival advantage in resected CRC patients with stage III or high-risk stage II disease [7, 8]. Cytotoxic chemotherapeutic agents inhibit the proliferation of cancer cells, but, at the same time, they produce toxicity and side effects on other tissues and organs. How to effectively and efficiently avoid or to alleviate the toxicity and side effects is a critical clinical problem that should be solved.

CID is a common side effect of the digestive system in the antitumor treatment process of cytotoxic drugs. The typical clinical characteristics of CID are as follows: symptoms range from loose stool without pain to severe, watery diarrhea with abdominal pain; symptoms typically last 5 to 7 days and may occur during or after chemotherapy; and patients with CID have a poor treatment response to gentamicin, berberine, and furoxone [9]. CID can lead to weakness, electrolyte disorders, renal failure, blood volume reductions, shock, and even death [10]. Its occurrence can result in delaying chemotherapy, increasing hospitalization time and costs, aggravating the psychological stress of patients, reducing the treatment compliance of patients and even altering the entire chemotherapy plan [11].

A unique microecosystem and characteristic microbial community lives in the colorectal regions owing to their function of storing feces [12]. Accumulating evidence points to the gut microbiome being involved in CRC [13]. For example, some researchers have reported that *Streptococcus bovis* [14] and *Streptococcus gallolyticus* [15] are specific bacteria involved in colorectal cancer; Castellarin et al. [16] found that *Fusobacterium nucleatum* infection is prevalent in CRC tissue specimens and Kostic et al. [17], found that *Fusobacterium nucleatum* can generate a proinflammatory microenvironment that is conducive to the progression of CRC through recruitment of tumor-infiltrating immune cells. Moreover, many enzymes, peptides and small molecules secreted by the intestinal gut microbiome are involved in activating and regulating important signaling molecules and signaling pathways involved in the progression of CRC [18, 19].

Diarrhea and gut microbiome disorders interact as both cause and effect. Diarrhea can disrupt the balance of the gut microenvironment. Meanwhile, the invasion of exogenous pathogenic microorganisms and the imbalance of intestinal microbes can lead to diarrhea [20]. For example, the invasion of some pathogenic bacteria including *Shigella*, *Salmonella* and *Klebsiella* [21, 22], and the abundance changes of intestinal parasitic microbes include *Candida albicans*, *Escherichia coli*, and *Aeromonas* can induce diarrhea [23–25]. Chemotherapy has been widely used in the clinic as an effective therapy for colorectal cancer, but diarrhea caused by chemotherapeutic drugs often affects the implementation of chemotherapy regimens [26]. Many probiotics and antibiotics play a positive role in the treatment of infectious diarrhea by regulating the gut microbiome [27, 28]. The study of relationships between gut microbiome and CID may provide a new direction for solving this clinical problem.

In the present study, we aimed to explore the association between the gut microorganisms and CID of the CapeOX regimen in resected stage III CRC and to provide some research methods and research ideas for further exploring the relationship between intestinal microbes and CID. The results may provide a fresh approach to the prevention and treatment of CID from a microbiological perspective.

## Subjects and methods

### Subjects

Patients with CRC treated at Huzhou Central Hospital from January 2016 to January 2018 were studied. The clinical protocols involving the patients and the informed consent form were approved by the Ethics Committee of Huzhou Central Hospital (No. 201601023). According to the American Joint Committee on Cancer (AJCC) staging manual system, stage III CRC is defined as when colorectal cancer is diagnosed by pathology with lymphatic metastasis and without distant metastasis [29]. The inclusion criteria were as follows. ① Cancer was confirmed by pathologic diagnosis, and the patients volunteered to participate in the study. ② The clinical stages conformed to the criterion of stage III CRC according to AJCC. ③ Patients voluntarily accepted and completed 8 cycles of the CapeOX regimen [capecitabine (1000 mg/m^2^ twice daily) combined with oxaliplatin (130 mg/m^2^ every 3 weeks)]. The exclusion criteria were as follows. ① Patients with diarrhea before chemotherapy. ② Patients with other intestinal diseases, such as ulcerative colitis and Crohn’s disease. ③ Alternation of chemotherapy regimen and chemotherapeutic dosage or the occurrence of progression of the disease (PD) during the 8 cycles of the CapeOX regimen. ④ Patients who accepted neo-adjuvant chemotherapy. ⑤ Patients with a history of the use of oral microbial agents within 1 month before chemotherapy. ⑥ Patients with another primary cancer. ⑦ Patients with known primary organ failure. ⑧ Patients who could not obtained the stool samples (n = 15) or obtained unqualified stool samples. All of the subjects signed informed consent under the guidelines approved by the Ethics Committee of Huzhou Central Hospital. CRC patients with and without CID were including in the experimental group and control group, respectively.

According to National Cancer Institute Common Toxicity Criteria (NCI CTC) V3.0 [30] CID grade 1 is defined as the number of defecations increased to less than 4 times a day and an excretion volume slightly increased. During a course of 8 cycles of chemotherapy administered over nearly half a year, there are many factors that can cause diarrhea, and these factors interfere with the diagnosis of CID. Clinical intervention measures, such as using antibiotics and antidiarrheal drugs, may be performed and the chemotherapy regimen may be changed in patients with CID grade 3 and grade 4. Therefore, we did not include patients with grade 1, grade 3 and grade 4 in the experimental group. CID grade 2 is defined as the number of defecations increased to 4–6 times per day or nocturnal stools, with the amount of excretion increased but with no interference in daily life. All of the subjects recruited into the experimental group were CID grade 2 in the present study.

### Collection of clinical data and stool samples

Basic information and clinical serological indicators were obtained from the medical record management system of Huzhou Central Hospital with informed consent from patients. Stool samples of the patients were collected in the 2 weeks after the 8 cycles of chemotherapy. Stool samples were collected in the morning prior to breakfast. An approximately 5–10 g stool sample was collected after defecation without the use of a purgative or lubricant. Within half an hour, the stool samples were stored in an ultra-low temperature freezer. The sample preservation time was not beyond 1 month. Finally, 17 stool samples from CRC patients were analyzed after the patients had signed informed consent forms and the unqualified specimens were eliminated.

### Intestinal microorganism detection

#### DNA extraction and PCR amplification

A E.Z.N.A.^®^ Stool DNA Kit (Omega Bio-Tek, Norcross, GA, U.S.) was used to extract total DNA from the stool samples according to the manufacturer’s protocols. The nanodrop ND-1000 spectrophotometer (LabTech, Washington, DC, USA) with the absorbances at 260 nm and 280 nm (A260/A280) was used to determine the quality and quantity of the purified DNA. DNA integrity was further verified by electrophoresis through a 2.0% (w/v) agarose gel. PCR (95 °C for 3 min, followed by 25 cycles at 95 °C for 30 s, 55 °C for 30 s, and 72 °C for 45 s and a final extension at 72 °C for 5 min) was used to amplify the V3–V4 region of the bacterial 16S ribosomal RNA gene (the primers of 16S V3–V4 rDNA are as follows: forward, CCTACGGGNGGCWGCAG and reverse, GACTACHVGGGTATCTAATCC). PCR amplifications were performed in triplicate in a 25 μL mixture containing 5 μL of DNA template, 2 μL of Nextera XT Index Primer 1 (10 M), 2 μL of Nextera XT Index Primer 2 (10 M) and 16 μL ddH_2_O. Amplicons were extracted from 2% agarose gels, purified using the AxyPrep DNA Gel Extraction Kit (Axygen Biosciences, Union City, CA, USA) and quantified using QuantiFluor™-ST (Promega, U.S.) according to the manufacturer’s instructions.

#### Library construction and sequencing

The MiSeq library was constructed as follows: purified PCR products were quantified by Qubit^®^ 3.0 (Life Technologies, Invitrogen). PCR products were ligated with Y adapter. Magnetic nanoparticles were used to take out the self-ligated Y adapters. An Illumina Pair-End library was constructed using pooled DNA products, and the amplicon library was pair-end sequenced (2 × 250) on an Illumina MiSeq platform (Shanghai BIOZERON Co., Ltd.) according to standard protocols (MiSeq V3 or V4 sequencing kit, Hua Ya Regenerative Medicine Biological Engineering Technology CO., LTD, Shanghai, China).

#### Sequencing data bioinformatics analysis

Sequencing data processing and optimization were performed according to the criteria at http://en.wikipedia.org/wiki/Fastq. Cutadapt (version 1.11) was used to demultiplex and quality filter the raw fastq files. Pandaseq (version 2.9) was used to assemble PE reads; only sequences that overlapped longer than 10 bp were assembled according to their overlap sequence. Mosaic sequences and sequences longer than 300–480 bp were discarded. In addition, the reads that receiving an average quality score < 20 were discarded.

The Silva database was used for comparison of 16S rRNA gene sequences [31] and the GeneBank database was used to analyze functional genes [32]. Mothur software [33] (http://www.mothur.org/wiki/Main_Page) was used to analyze the microbial diversity. OTU (operational taxonomic units), as the artificial classification unit, was used to estimate the number of species in each sample, and the similarity threshold was 97%. The coverage (http://www.mothur.org/wiki/Coverage) was used as the sequencing depth index. The ACE estimator (http://www.mothur.org/wiki/Ace) and Chao estimator (http://www.mothur.org/wiki/Chao) were used to estimate the gut microbial community richness. The Shannon index (http://www.mothur.org/wiki/Shannon) and Simpson index (http://www.mothur.org/wiki/Simpson) were used to estimate the gut microbial community diversity. The pie charts were used to describe the distribution of microorganisms at the species level. Proportions less than 1% were included in “Others”, considering the best visual presentation. Venn diagrams were implemented by Venn diagram. We performed clustering on genera obtained from the RDP Classifier by means of the complete linkage hierarchical clustering technique using the R package HCLUST (http://sekhon.berkeley.edu/stats/html/hclust.html). The basic process of LDA analysis [34] was as follows: First, non-parametric Kruskal–Wallis rank sum test was used to detect the significant difference of abundance among species between the two groups of samples. Then Wilcoxon rank sum test was used to analyze the difference between groups based on the significant difference species. Finally, LDA was used to reduce the dimension of data and evaluate the influence of significantly different species (LDA score). LDA effect size more than 2 was included in the figure. The genes of the 16S corresponding species and their copy number have been generally known, and these genes can be compared with databases (e.g. KEGG, NOG) to predict the possible metabolic capacity of these species. The Picrust software was used to analyze the pathway based on the KEGG database. The specific methods of function prediction analysis are shown in the following website (http://picrust.github.io/picrust/tutorials/algorithm_description.html).

#### Statistical analysis

SPSS software (V: 16.0; SPSS Inc., Chicago, IL) was used to analyze the data. The data are expressed as the mean ± standard deviation (SD). Statistically significant differences between two groups were analyzed using the independent-samples t-test. Frequency table data were analyzed with the Chi-square test or Fisher’s exact test. All tests were performed at the 0.05 level of significance.

## Results

### Basic characteristics of the patients

**Fig. 1:**
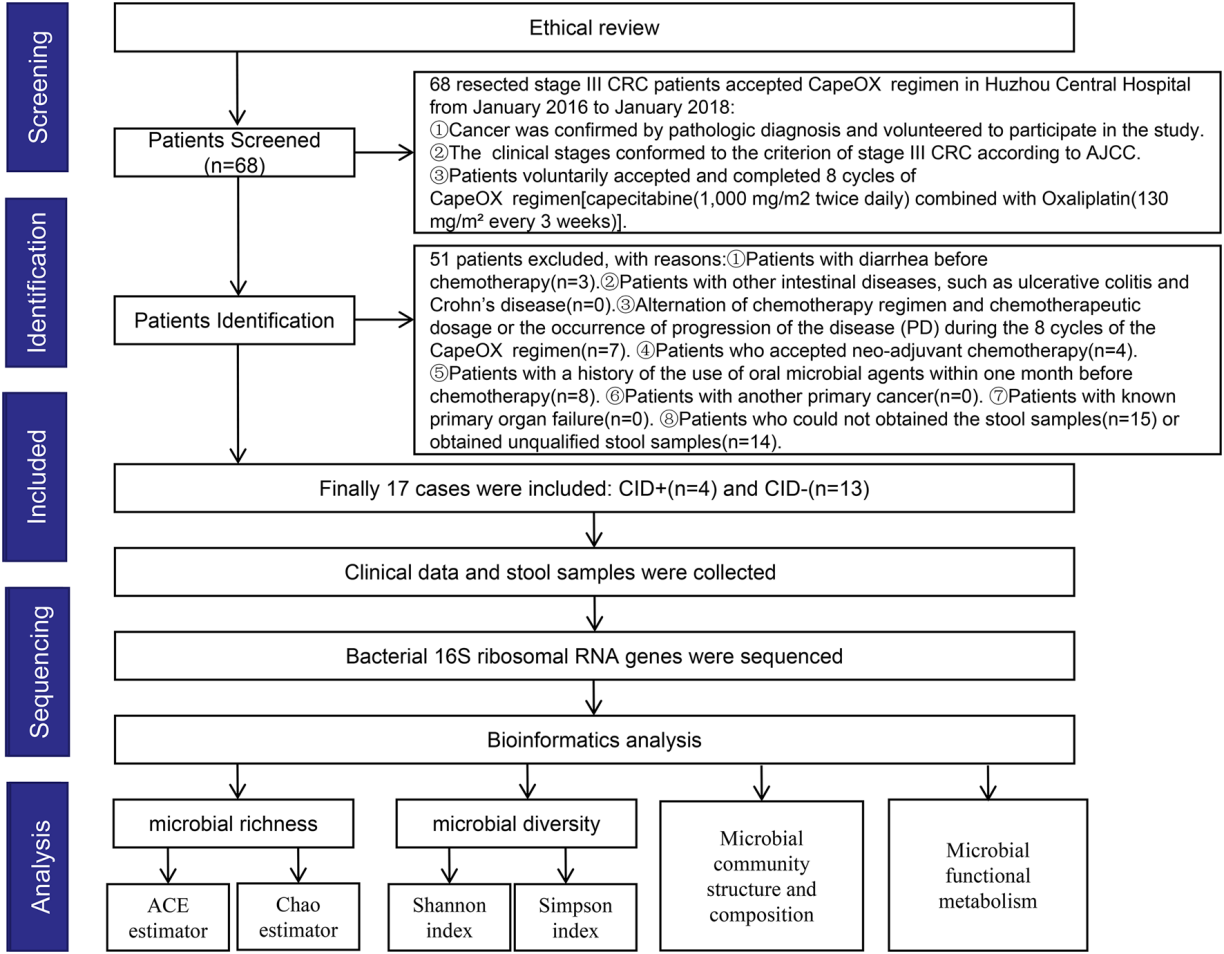
Study strategy. After screening and identification, finally, 17 stage III CRC patients undergoing the CapeOX regimen after curative resection were recruited into the present study. Stool samples were collected from the recruited patients. The bacterial 16S ribosomal RNA from the samples were sequenced. Bioinformatics analysis was used to analyze the distinctive gut microbiome and functional metabolism

The clinical stages conformed to the criterion of stage III CRC according to AJCC. Overall, 68 resected stage III CRC patients accepted the CapeOX regimen in Huzhou Central Hospital from January 2016 to January 2018. However, 51 patients were excluded from this study for the following reasons: patients with diarrhea before chemotherapy (n = 3), terminated chemotherapy because of PD and side effects (n = 7), accepted neo-adjuvant chemotherapy (n = 4), could not obtain the stool samples (n = 15), unqualified stool samples (n = 14), and used oral probiotics including Bifid-triple Viable Capsule (n = 6) and Clostridium Butyricum Capsule (n = 2) before chemotherapy. After screening and identification, finally, 17 stage III CRC patients were enrolled in the present study. The study strategy is shown in Fig. 1. The pathology type of all of the patients with resected stage III colorectal cancer was moderately differentiated adenocarcinoma. Smoking and drinking history over the course of 1 year were collected. The 17 CRC patients were divided into experimental group (CID+) and control group (CID−) according to whether they had diarrhea or not. The patient’s clinical information is shown in Table 1. The clinical stages conformed to the criterion of stage III CRC according to AJCC. Smoking and drinking history over the course of 1 year were collected. Blood samples of patients before chemotherapy were collected for serological examination. There was no significant difference between the two groups in terms of age, sex, BMI, or smoking, drinking history and clinical serological indicators including hemoglobin, alanine transaminase, glutamic oxaloacetic transaminase, albumin, creatinine, carbohydrate antigen 199, carbohydrate antigen 742 and carcino embryonic antigen.

**Table 1:** Characteristics of the study participants

	Experimental group	Control group	p value
Cases, n	4	13	–
Rectal cancer, n	2	6	0.89
Males, n	3	8	0.62
Age (years)	63.88 ± 8.61	64.23 ± 8.76	0.94
BMI (kg/m^2^)	22.74 ± 2.64	23.28 ± 2.43	0.71
Long-term smoking history, n	0	1	0.57
Long-term drinking history, n	1	4	0.82
Known diabetes, n	0	1	0.57
Known hypertension, n	0	0	/
Hemoglobin (g/L)	134.25 ± 5.06	124.62 ± 13.71	0.20
Alanine transaminase (U/L)	29.00 ± 30.97	19.80 ± 8.94	0.33
Glutamic oxaloacetic transaminase (U/L)	27.40 ± 15.78	24.56 ± 8.72	0.64
Albumin (g/L)	42.03 ± 4.57	38.11 ± 3.02	0.06
Creatinine (μmol/L)	83.10 ± 33.74	68.95 ± 14.66	0.24
Carbohydrate antigen 199 (U/mL)	5.46 ± 3.61	12.24 ± 9.36	0.18
Carbohydrate antigen 742 (U/mL)	8.58 ± 3.61	9.89 ± 19.71	0.90
Carcino embryonic antigen (ng/mL)	24.34 ± 41.47	3.98 ± 3.70	0.08

Overall, 17 resected stage III CRC patients undergoing the CapeOX regimen were recruited. These patients were divided into experimental group (CID+) and control group (CID−) according to whether they had diarrhea or not. The patient’s clinical information was shown in thus table. The clinical stages conformed to the criterion of stage III CRC according to AJCC. Smoking and drinking history over the course of 1 year were collected. Blood samples of patients before chemotherapy were collected for serological examination

### Gut microbial richness and diversity associated with CID during the Cape OX regimen for CRC

As shown in Table 2, the coverage index suggested that the coverage of the genomic library of the sequencing samples was more than 99%. The statistically significant differences of the ACE estimator and Chao estimator between the experimental group and control group indicated that the community richness of the gut microbiome was lower in the patients with chemotherapy-induced diarrhea (p < 0.05, vs control group). The smaller Shannon index and larger Simpson index indicate that the gut microbial community diversity was lower in the patients with chemotherapy-induced diarrhea (p < 0.01, vs control group). The alpha diversity analysis is shown in Additional file 1.

**Table 2:** Gut microbial richness and diversity associated with chemotherapy-induced diarrhea of the CapeOX regimen in CRC

Group	n	Coverage	OTU	Ace	Chao	Shannon	Simpson
Experimental group	4	0.9994 ± 0.0008	119.75 ± 53.44	159.50 ± 100.09	150.75 ± 77.58	2.21 ± 0.47	0.21 ± 0.08
Control group	13	0.9985 ± 0.0012	273.38 ± 93.68	336.31 ± 117.73	332.46 ± 113.34	3.36 ± 0.59	0.09 ± 0.07
t value	–	–	− 3.08	− 2.70	− 2.97	− 3.54	2.30
p value	–	–	0.008	0.016	0.010	0.003	0.009

Mothur software (http://www.mothur.org/wiki/Main_Page) was used to analyze the microbial diversity. Operational taxonomic units (OTU), as the artificial classification unit, was used to estimate the number of species in each sample, and the similarity threshold was 97%. The alpha diversity analysis was shown in Additional file 1

### Gut microbial community structure associated with CID during the CapeOX regimen for CRC

**Fig. 2:**
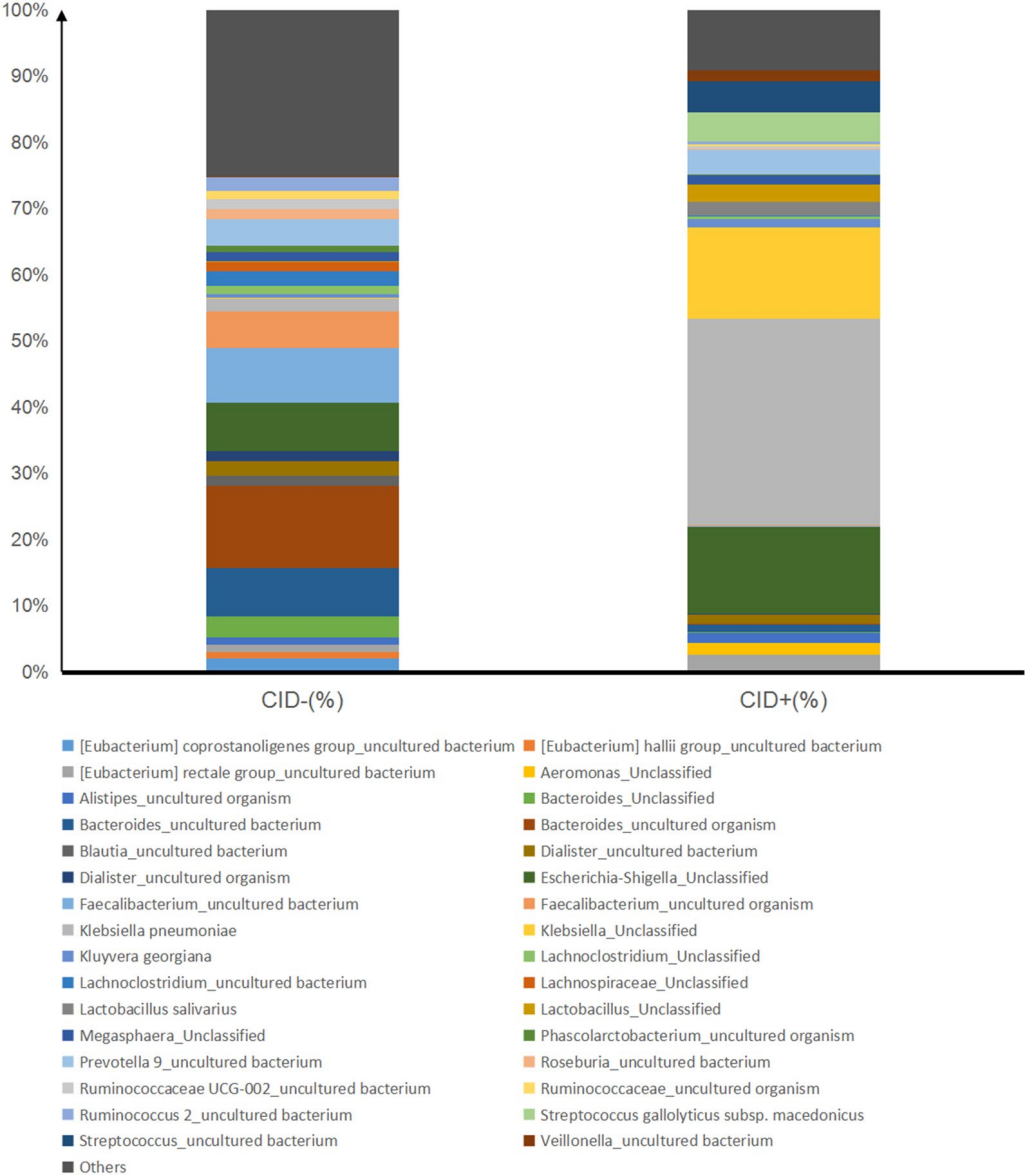
Microbial community structure analysis. The distribution of microorganisms from the same group was merged into the whole. The stacked bar graph was used to describe the distribution of microorganisms at the species level. CID- and CID+ represent the control group and experimental group, respectively

A pie chart was constructed for the microorganisms that accounted for more than 1% in the two groups of samples to show the microbial community structure associated with CID during the CapeOX regimen for CRC. The distribution of microorganisms from the same group was merged into a whole. As shown in Fig. 2, the stacked bar graph showed that the percent of *Klebsiella pneumoniae* was 35% and it occupied the highest proportion in the experimental group (CID+). But the rate was only 3% in the control group (CID−).

### The difference in microbial abundance associated with CID of the CapeOX regimen for CRC

**Fig. 3:**
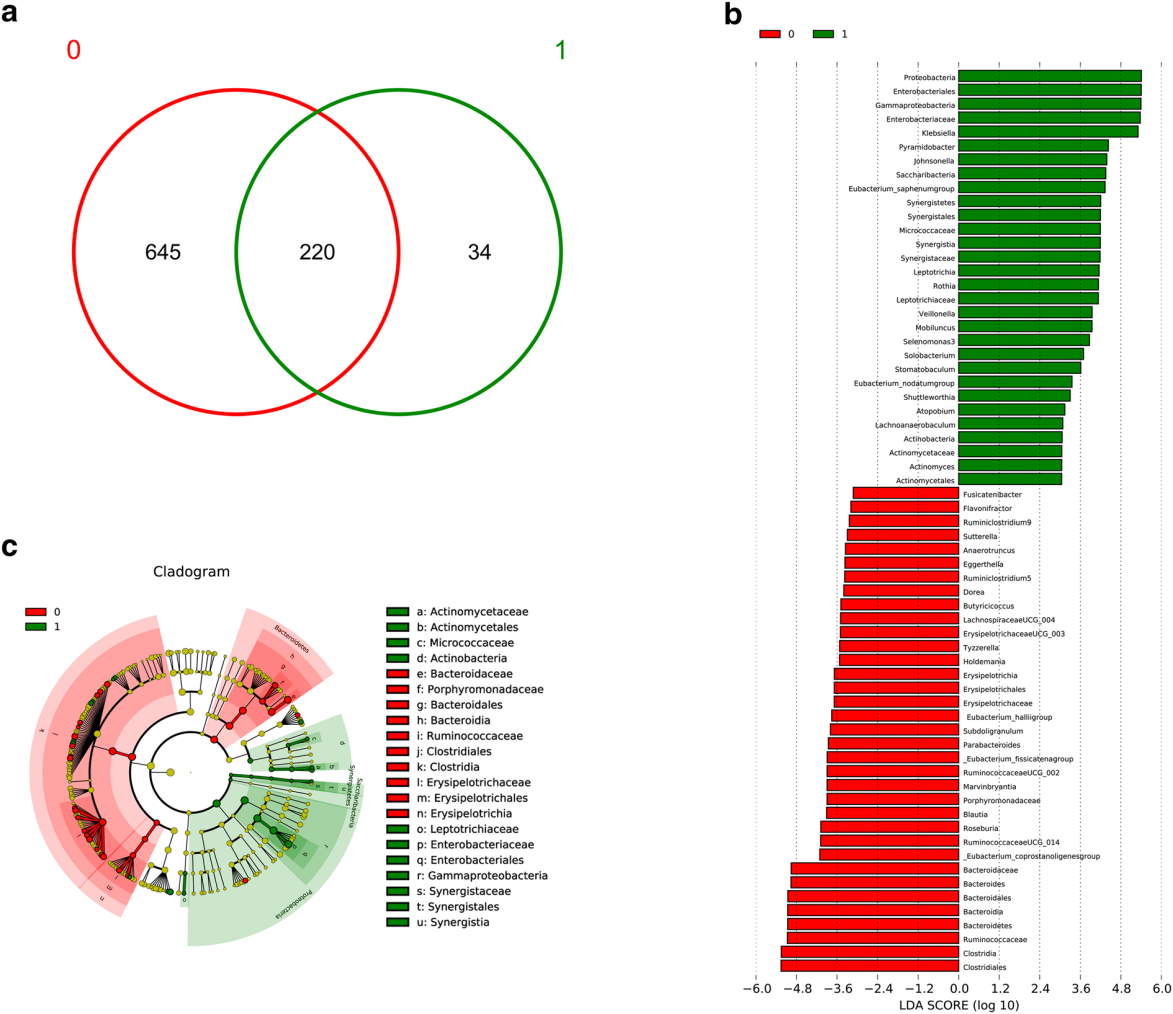
Differential microbes analysis. The samples were divided into two groups according to patients with or without CID. The number of OTUs that were exclusive or shared by the two groups were visualized by a Venn Chart (**a**). The strip figure in **b** displays the LDA effect size analysis. LDA effect size analysis was used to compare the differences in microbial abundance between the two groups (**b**). LDA scores over 2 mean that the differences are statistically significant (p < 0.05). The cladogram in **c** displays the composition and proportion of microorganisms at different taxonomic levels. The innermost layer shows the taxonomic tree. The circle from the inside to the outside represents different taxon levels from phylum to genus. Yellow circles represent no statistical difference in species between the two groups, and red circles and green circles represent the higher abundance of genus in the control group and the chemotherapy-induced diarrhea group, respectively. The lowercase English letters corresponding to the different bacteria are shown in the legends

The samples were divided into two groups according to patients with or without CID. As shown in Fig. 3a, there were 220 shared OTUs and that the numbers of unique OTUs in the control group and experimental group were 645 and 34, respectively. LDA effect size analysis was used to compare the differences in microbial abundance between the two groups. LDA Effect Size analysis (Fig. 3b) showed that there were 75 microorganisms with significant differences at the genus level between the two groups. The length of the histogram indicates that some microorganisms such as *Proteobacteria*, *Enterobacteriales*, *Gammaproteobacteria*, *Enterobacteriaceae*, *Klebsiella*, *Clostridiales*, *Clostridia*, *Ruminococcaceae*, *Bacteroidetes*, *Bacteroidia*, *Bacteroidales*, *Bacteroides and Bacteroidaceae* have a larger influence. The cladogram in Fig. 3c displays the composition and proportion of microorganisms at different taxonomic levels. The cladogram showed that the abundance of these bacteria including *Actinomycetaceae*, *Micrococcaceae*, *Bacteroidaceae*, *Porphyromonadaceae*, *Ruminococcaceae*, *Erysipelotrichaceae*, *Leptotrichiaceae*, *Enterobacteriaceae* (*Klebsiella* belongs to *Enterobacteriaceae*) and *Synergistaceae* had significant differences at family level between the two groups.

### The functional analysis of 16S rRNA genes associated with CID during the CapeOX regimen for CRC

**Fig. 4:**
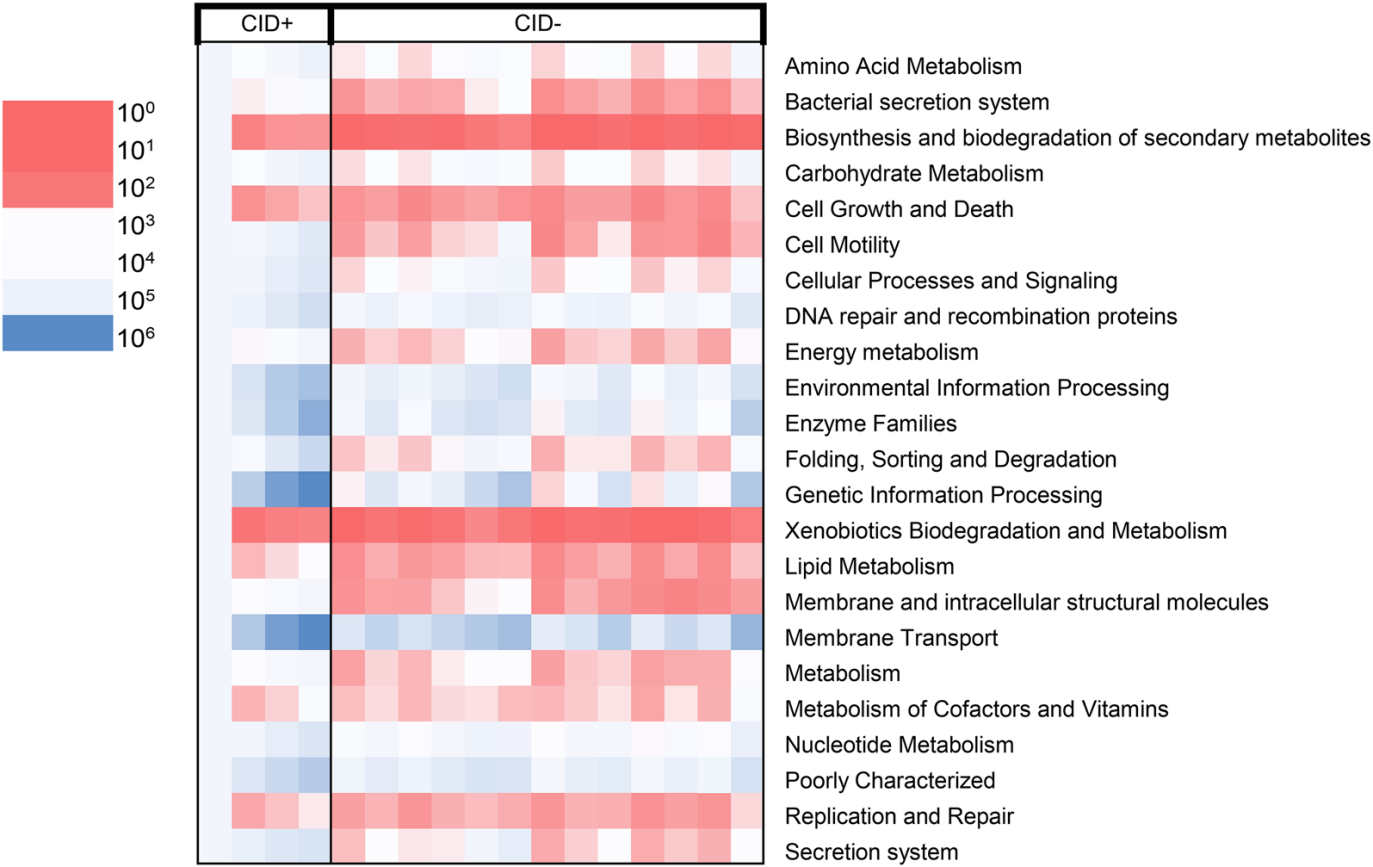
Functional analysis. The Picrust software was used to analyze the pathway based on the KEGG database. The significant differences in functional metabolism are shown in the figure

It has been confirmed that many gene function annotation databases are associated with corresponding 16S rRNA gene sequences. Functional analysis of enzymes and genes would be conducive to understand the whole microecological environment. As shown in Fig. 4, the figure shows the results of the functional analysis. Overall, 23 pathways (p < 0.001) associated with statistically significant differences might be involved in CID during the CapeOX regimen for adjuvant chemotherapy in resected stage III colorectal cancer. These differential pathways are related to microbial metabolites, cell proliferation and death, immune system, and many other aspects.

## Discussion

The CapeOX regimen is recommended as the postoperative adjuvant chemotherapy regimen in resected CRC patients with stage III or high-risk stage II [35]. The high-risk factors in stage II may affect the accuracy of experimental results, Thus, CRC patients with stage III were recruited in the present study. According to the NCCN guidelines, the CapeOX regimen for resected stage III CRC has been defined as 8 cycles of capecitabine (1000 mg/m^2^ twice daily) combined with oxaliplatin (130 mg/m^2^ every 3 weeks) [36]. CID is one of the most common side effects of the CapeOX regimen [37]. The degree of diarrhea caused by different chemotherapeutic agents varies. Among the chemotherapeutic drugs for colorectal cancer, the most common drugs that cause diarrhea are fluorouracil, irinotecan and platinum, and the incidence of diarrhea associated with these drugs can be as high as 50–80% [38]. Our previous clinical investigation found that the probability of CID increased with an increase of chemotherapeutic frequency and chemotherapeutic dosage and most cases of CID occur within 2 weeks after chemotherapy. Therefore, in the present study, stool samples from the patients were collected in 2 weeks after the 8 cycles of chemotherapy of the CapeOX regimen were completed.

Studies of Carroll et al. [39] suggested that the richness of 16S rRNA sequences was significantly decreased in patients with diarrhea-predominant irritable bowel syndrome. Lee [40] has reported the community diversity of gut microbiome is lower in patients with kidney posttransplant diarrhea. Similar results have been reported in dogs with acute diarrhea [41]. The research on these diarrhea diseases is consistent with our findings. We found that the gut microbial community richness and community diversity was lower in CRC patients with CID in the present study. The decrease of microbial diversity may be related to the imbalance of gut microbiome. The dominant pathogenic bacteria leads to the reduction of the resident normal microbiota through the plunder of nutrients or the killing effect of bacterial toxic metabolites.

Our results from the microbial community structure analysis showed *Klebsiella pneumoniae* occupied the highest proportion (31.22%) of gut microbiome in CRC patients with CID. Lu et al. [42] reported that the rate of *Klebsiella pneumoniae* was approximately 0.5% in Beijing by culturing the stool pathogens from outpatients with diarrhea syndromes using the Vitek2 Compact instrument. Zhang et al. [22] only isolated 43 *Klebsiella pneumoniae* strains from 551 stool specimens from diarrhea patients. Although the study may have be interfered by other factors such as geographical location, nosocomial infection, or pollution contamination in the fecal collection process. The increased proportion of *Klebsiella pneumoniae* in patients with CID remains a phenomenon of concern.

Microorganisms can adjust and modify their surroundings due to their metabolic products. A series of enzymes and genes participate in the process of metabolism [12]. Functional analysis of these enzymes and genes would be conducive to understand the whole microecological environment. In the present study, we screened 75 differentiated microorganisms at the species level by using LDA effect size analysis as well as 23 pathways associated with differential microorganisms by using KEGG databases. These differential pathways are related to microbial metabolites, cell proliferation and death, immune system, and many other aspects. These differentiated microorganisms, their metabolic products and the relevant pathways make up the intestinal microecosystem that causes CID. These results may provide characteristic microorganisms or potential molecular targets for the treatment and prevention of CID.

It must be emphasized that small sample size and potential sample contamination may affect the accuracy of the present study. Strict inclusion and exclusion criteria was performed in this study. Subjects in the experimental group were only included the resected stage III colorectal cancer (CRC) patients who voluntarily accepted and completed 8 cycles of the CapeOX regimen and accompanied with grade 2 of CID. Thus, the small sample size limits the applicability of the findings. A multi-center, large study will provide more powerful data to support the clarification of the microbial differences in CRC patients with CID.

## Conclusion

Chemotherapy induced diarrhea (CID) is a common side effect in patients receiving chemotherapy for cancer. In the present study, We found that the gut microbial community structure and community diversity have changed in CRC patients with CID. The gut microbial community richness and community diversity were lower in CID. *Klebsiella pneumoniae* was the most predominant species among the gut microbiome in CRC patients with CID. It may provide novel insights into the prevention and treatment of patients with CID to improve patients’ quality of life and chemotherapy tolerance.

## Additional file

**Additional file 1.** Alpha diversity analysis.

## Abbreviations

CRC: colorectal cancer
AJCC: American Joint Committee on Cancer
NCCN: National Comprehensive Cancer Network
OTU: operational taxonomic unit
PD: progression of disease
PFS: progression free survival
OS: overall survival
mCRC: metastatic colorectal cancer
CID: chemotherapy-induced diarrhea
NCI: National Cancer Institute
CTC: common toxicity criteria
