# Supplementary material for: Gut microbiome associated with chemotherapy-induced diarrhea from the CapeOX regimen as adjuvant chemotherapy in resected stage III colorectal cancer
Source: Gut Pathog. 2019 Apr 30;11:18. doi: 10.1186/s13099-019-0299-4 (PMC6489188; doi:10.1186/s13099-019-0299-4)
Supplement: Supplementary file 1 — Additional file 1. Alpha diversity analysis. [file 13099_2019_299_MOESM1_ESM.docx]

Additional Data. Alpha Diversity Analysis

Experimental group(CID+) includes C6, C8, C13 and C15.

Control group(CID-) includes C1-5,C7,C9-12, C14 and C16-17.


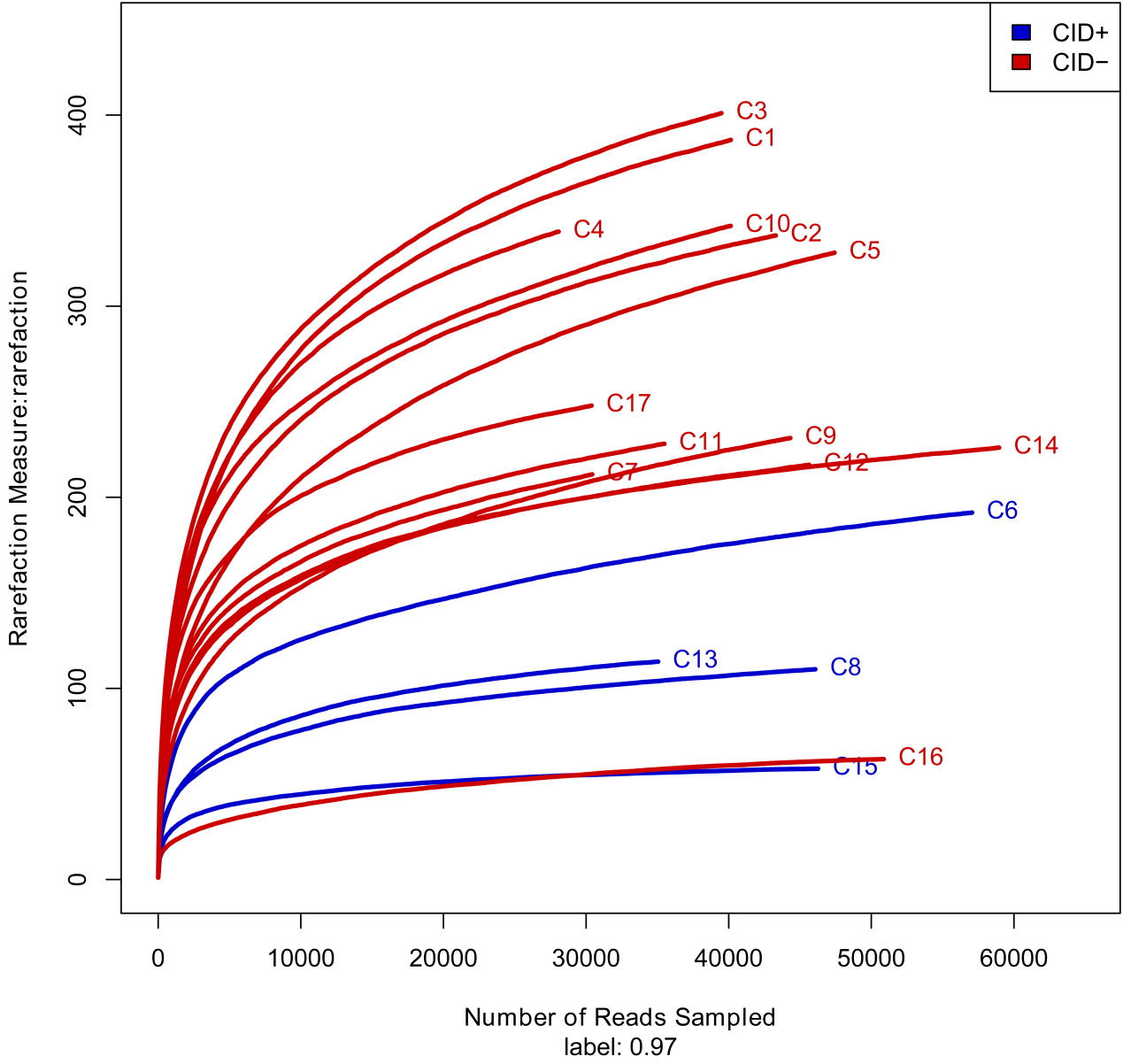


Figure S1. The rarefaction curve for each sample

Rarefaction curve is a plot of the number of OTU as a function of the number of sequencing reads. The species richness can be compared among samples with different sequencing depth by applying the rarefaction curve. The flattening of the rarefaction curve indicates that the sequencing depth is appropriate.


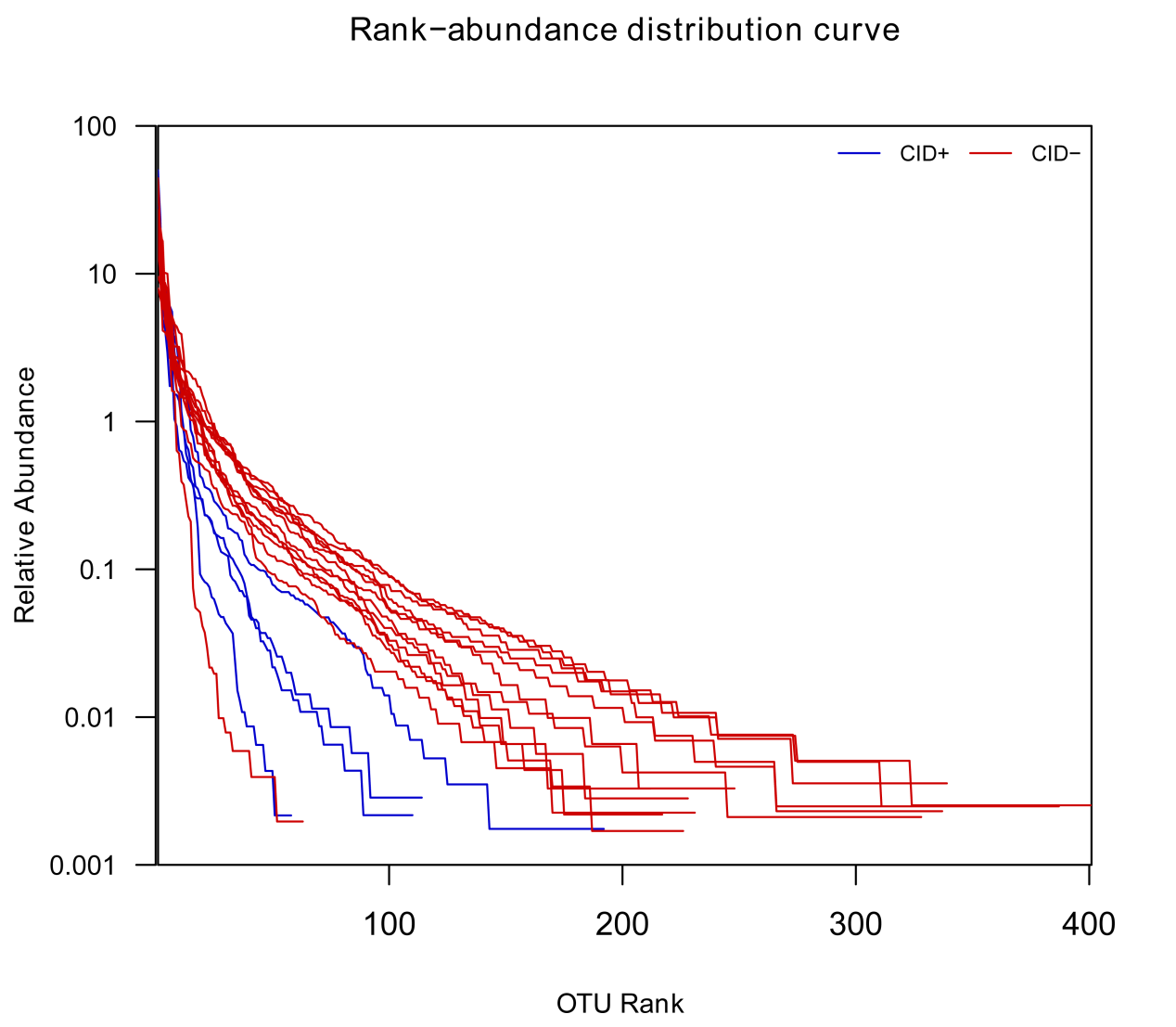


Figure S2. OTU Rank-Abundance curves

Rank-abundance is utilized for evaluating the microbial diversity of the sample. The sequence number of each OTU is plotted against the rank of the OTU (the more sequences the OTU has, the smaller rank number is assigned to the OTU). The statistical differences between the groups were shown in **Table 2.**


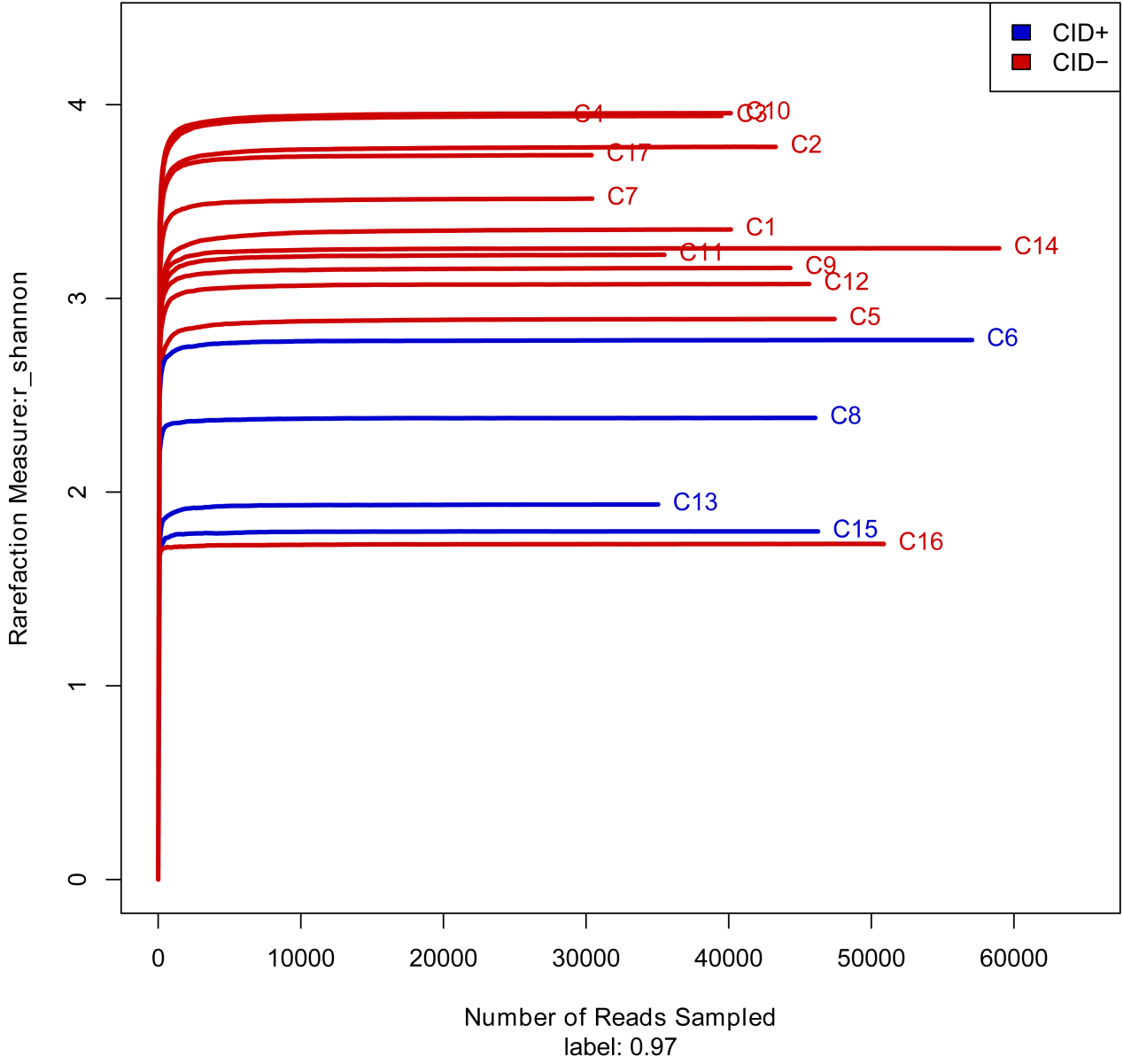


Figure S3. Shannon Wiener curves of samples.

Shannon-Wiener index indicates the level of microbial diversity of the sample. The statistical differences between the groups were shown in **Table 2**.
